# Impact of Dysfunctional Feed-Forward Inhibition on Glutamate Decarboxylase Isoforms and γ-Aminobutyric Acid Transporters

**DOI:** 10.3390/ijms22147740

**Published:** 2021-07-20

**Authors:** Sandesh Panthi, Nikita M. A. Lyons, Beulah Leitch

**Affiliations:** Department of Anatomy, School of Biomedical Sciences, Brain Health Research Centre, University of Otago, Dunedin 9016, New Zealand; sandesh.panthi@otago.ac.nz (S.P.); nikita.lyons@otago.ac.nz (N.M.A.L.)

**Keywords:** stargazers, absence epilepsy, GAD isoforms, GABA transporters, feed-forward inhibition, inhibitory DREADD

## Abstract

Absence seizures are associated with generalised synchronous 2.5–4 Hz spike-wave discharges causing brief and sudden alteration of awareness during childhood, which is known as childhood absence epilepsy (CAE). CAE is also associated with impaired learning, psychosocial challenges, and physical danger. Absence seizures arise from disturbances within the cortico-thalamocortical (CTC) network, including dysfunctional feed-forward inhibition (FFI); however, the precise mechanisms remain unclear. In epileptic stargazers, a genetic mouse model of CAE with chronic seizures, levels of γ-aminobutyric acid (GABA), and expression of GABA receptors are altered within the CTC network, implicating altered GABAergic transmission in absence seizures. However, the expression of GABA synthesising enzymes (GAD65 and GAD67) and GABA transporters (GAT-1 and 3) have not yet been characterised within absence seizure models. We found a specific upregulation of GAD65 in the somatosensory cortex but not the thalamus of epileptic stargazer mice. No differences were detected in GAD67 and GAT-3 levels in the thalamus or somatosensory cortex. Then, we assessed if GAD65 upregulation also occurred in Gi-DREADD mice exhibiting acute absence seizures, but we found no change in the expression profiles of GAD65/67 or GAT-3. Thus, the upregulation of GAD65 in stargazers may be a compensatory mechanism in response to long-term dysfunctional FFI and chronic absence seizures.

## 1. Introduction

Childhood absence epilepsy (CAE) is one of the most prevalent paediatric epilepsies, accounting for 10–17% of all diagnosed epilepsy cases in school-aged children [1,2]. Absence seizures are the clinical manifestation of CAE and are characterised by brief behavioural arrest, loss of awareness, and an electrographic signature of spike-wave discharges (SWDs) measuring 2.5–4 Hz on an electroencephalogram (EEG) [3,4]. These brief episodes of impaired consciousness may occur hundreds of times a day and can increase the chance of physical injury when undertaking activities such as swimming and cycling [5]. EEG and functional imaging studies suggest that absence seizures are likely due to aberrant activity within the cortico-thalamocortical (CTC) network [6,7], but the precise mechanisms are still unclear and likely to be multifactorial. Current treatment options for CAE are insufficient as up to 30% of patients are pharmaco-resistant, reporting intolerable side effects and/or seizure aggravation. Additionally, ≈60% of children with CAE have severe neuropsychiatric comorbid conditions including attention deficits, mood disorders, and impairments in memory and cognition [6]. Thus, safe, effective, and patient specific treatment approaches are imperative to successfully treat more patients, improving their quality of life. To develop novel therapeutic targets and discover new anti-epileptic drugs (AEDs), it is crucial to first understand the precise cellular and molecular mechanisms underlying absence seizures in different models of CAE.

A lack of stargazin protein in the stargazer mouse model of absence epilepsy reduces the expression of α-amino-3-hydroxy-5-methyl-4-isoxazolepropionic acid receptors (AMPARs) at excitatory synapses onto feed-forward inhibitory (parvalbumin expressing; PV+) interneurons of the CTC network i.e., those in the somatosensory cortex (SScortex) [8,9,10,11] and reticular thalamic nuclei (RTN) of the thalamus) [12,13]. This deficit in stargazin, a transmembrane AMPAR transporting protein (TARP), impairs excitatory input to PV+ inhibitory interneurons, thus likely reducing FFI within the cortical and thalamic microcircuits of the CTC network. It is important to note that functional silencing of CTC feed-forward inhibitory PV+ interneurons is sufficient to elicit both electrographic and behavioural correlates of seizures in normal mice [14], whereas activating these interneurons during chemically induced absence seizures either reduces their severity or prevents absence seizure generation [15].

In the CTC network of epileptic stargazer mice, levels of GABA and its receptors (GABARs) show region-specific alterations. In the thalamus, GABA levels are significantly reduced in the ventral posterior (VP) nucleus [16] but not the RTN region. Conversely, phasic and tonic GABA_A_Rs are only increased in the VP region [17,18]. However, GABA_A_R upregulation in the stargazer VP thalamus occurs only after seizure onset at postnatal days (PN)17–18 [19]. This suggests a seizure-induced effect, potentially to compensate for a lack of GABA in the stargazer VP thalamus, with GABA_A_R upregulation likely contributing to seizure maintenance rather than generation. In the SScortex, by contrast, GABA transmitter levels are increased [16]. Interestingly, increased GABA levels have also been recorded in the affected cerebral hemisphere of a CAE patient with unihemispheric SWDs [20]. Thus, the GABAergic system dysfunction in animal models of absence epilepsy is likely recapitulating disturbances occurring in human patients. Altered GABA levels may be a future therapeutic target for CAE; however, the causative upstream molecular changes are not yet known.

GABA levels are controlled by the enzymes responsible for its production and transporters mediating its reuptake. Glutamate decarboxylases (GADs) synthesise GABA from glutamate and exist as one of two isoforms: GAD65 and GAD67. GAD65 is predominantly localised to axon terminals where it catalyses the on-demand GABA synthesis crucial for phasic inhibition [21]. On the other hand, GAD67 is distributed throughout GABAergic neurons, synthesising the majority of GABA, and it is important in mediating tonic inhibition [22,23].

GABA levels are also regulated by the GABA transporters (GATs: GAT-1 and GAT-3) that mediate its reuptake from the extracellular space. GAT-1 is expressed in neurons and some astrocytic processes, where it regulates GABA levels during sustained neuronal activity. Conversely, GAT-3 is produced primarily in astrocytes and regulates GABA levels in extrasynaptic areas [24,25].

In the genetic rat model of absence epilepsy (GAERS), absence seizures are thought to be caused by excess tonic inhibition in the VP thalamus due to insufficient GAT-1-mediated reuptake of extracellular GABA. Increased tonic GABAergic inhibition is evident in the VP thalamus of GAERS after PN17 and is sustained up to PN30 when SWDs first appear [26]. Increased tonic GABAergic inhibition also occurs in the VP thalamus of stargazers; however, this is not evident until PN19–21, after seizure onset. Thus, increased tonic inhibition is unlikely to be causative of seizures in the stargazer model of CAE.

In a recent study, GABA uptake assays were performed in primary cultures of astrocytes from the thalamus and cortex of GAERS. The uptake activity of each transporter was determined by selective pharmacological blockade showing functional alterations somewhat at odds with GAT expression changes. GAT-1 expression increased and GAT-3 expression decreased in GAERS thalamic astrocytes, which showed reduced GABA uptake by both GATs. Additionally, in GAERS cortical astrocytes, where GAT-3 mediated uptake was reduced, GAT-1 and GAT-3 were both upregulated [27]. Moreover, GAT-1 knockout mice show spontaneous ethosuximide (ETX)-sensitive SWDs [26] as well as physical and behavioural abnormalities [28,29], while blocking GAT-1 in normal non-epileptic Wistar rats generates absence seizures [26]. Thus, GAT-1 and/or GAT-3 may have a role in altering GABAergic inhibition and contributing to absence epilepsy.

Currently, less is known about the role of GADs in epilepsy. GAD65 knockout animals are highly vulnerable to seizures [30,31,32,33] and have a poor survival rate with 25% of GAD65 knockout mice dying by 6 months of age [33]. GAD65 knockout rat pups appear normal at birth; however, they begin to exhibit severe (generalised tonic-clonic) or mild (myoclonic) epileptic seizures during the third postnatal week and have an 80% mortality rate within PN23 [34]. In both studies, the increased lethality of GAD65 knockout animals was attributed to spontaneous seizures. On the other hand, GAD67 knockout is embryonic lethal [22,35], so less is known about its role in seizure genesis. Expression levels of GAD65 and GAD67 are reduced in human temporal lobe epilepsy patients [36], suggesting a potential role of GADs in epilepsy, which remains to be elucidated.

Clearly, there are many mechanisms that can impair normal GABAergic inhibition within the CTC network. Abnormalities in the expression of GADs and GATs may play a role in altering GABA levels and thus GABAergic inhibition within the CTC network, leading to absence seizures. However, to date, there have been no studies specifically analysing the relative expression levels of GADs and GATs within the CTC network of a genetic mouse model of absence epilepsy. Hence, in this study, we first aimed to investigate the expression profile of GADs and GATs in the stargazer mouse model of absence epilepsy, which is known to have altered GABA levels in the CTC network [16]. In this model, absence seizures occur chronically from early postnatal development (PN17–18 days) through adulthood [37] due to a genetic mutation that reduces AMPAR expression at input synapses to PV+ FFI interneurons. The second objective of this study was to determine if acute functional silencing of PV+ FFI interneurons (sufficient to cause absence seizures) in Gi-DREADD (Designer Receptors Exclusively Activated by Designer Drug) mice [14,15] alters the level of GADs and/or GATs and hence GABA levels over a short time scale. Recent evidence has shown rapid (within minutes) de novo synthesis of local proteins in presynaptic axon terminals participating in synaptic transmission [38]. Thus, acute Gi-DREADD mediated silencing may stimulate fast compensatory changes in GAD or GAT levels in response to reduced synaptic transmission.

## 2. Results

### 2.1. Expression Pattern of GADs and GATs in the SScortex and the Thalamus of Stargazers and Non-Epileptic Controls

**Figure 1:**
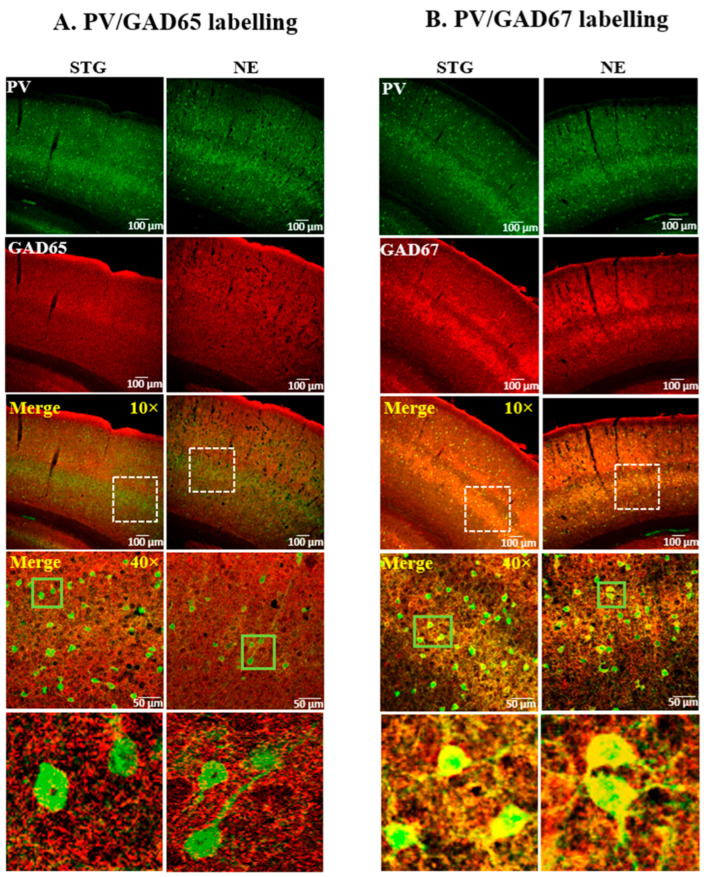
Representative confocal images showing the expression of (**A**) GAD65 or (**B**) GAD67, and PV in the SScortex of epileptic stargazers (STG) and non-epileptic littermates (NE). Merged images at low magnification (10×) and medium magnification (40×) are shown. White dotted boxes in the third panel were magnified in the fourth panel. Green boxes in the fourth panel were magnified in the bottom panels of figures (**A**,**B**) to show punctate structures and colocalisation, respectively.

**Figure 2:**
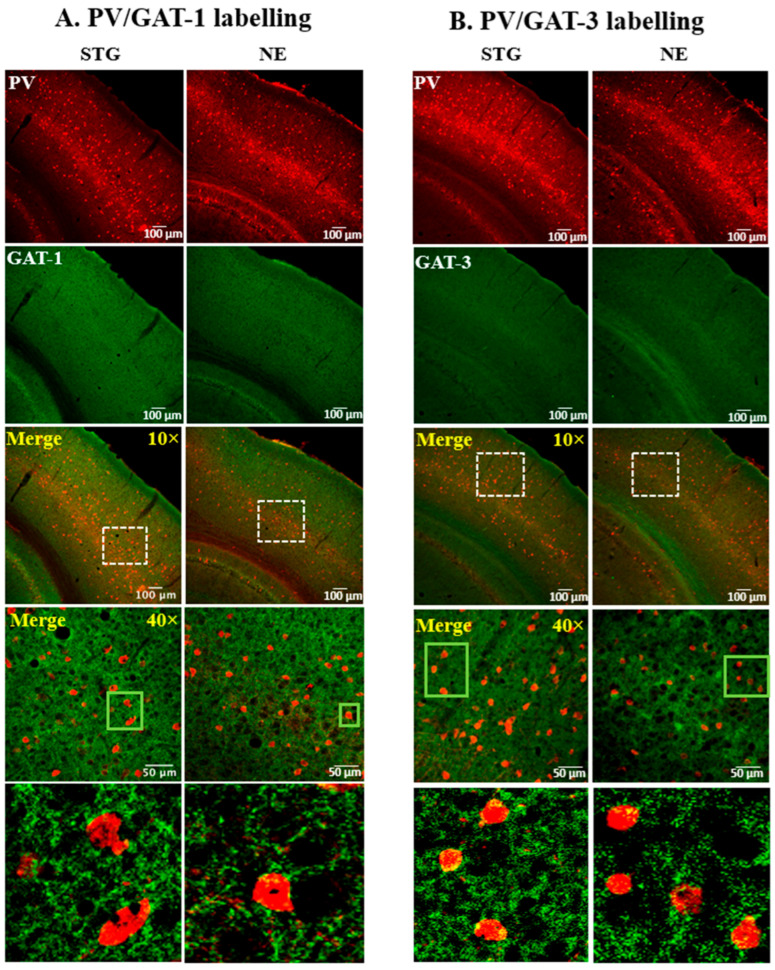
Representative confocal images showing the expression of (**A**) GAT-1 or (**B**) GAT-3 and PV in the SScortex of epileptic stargazers (STG) and non-epileptic littermates (NE). Merged images at low magnification (10×) and medium magnification (40×) are shown. White dotted boxes in the third panel were magnified in the fourth panel. Green boxes in the fourth panel were magnified in the bottom panels of figures (**A**,**B**) to show punctate structures.

**Figure 3:**
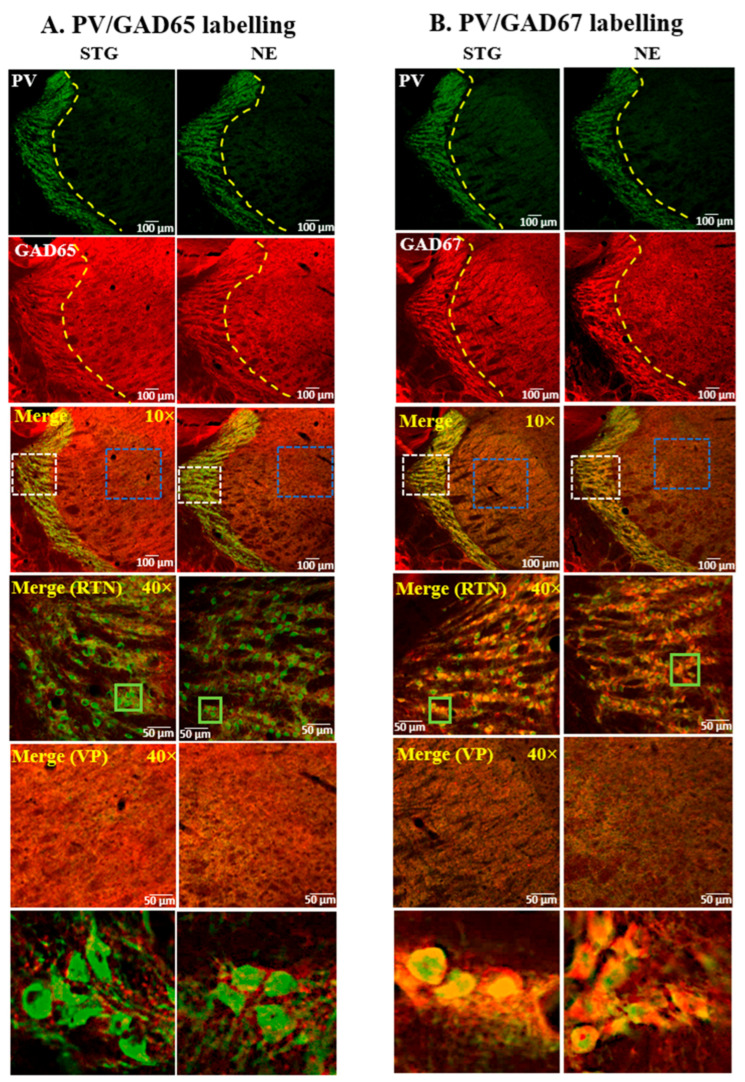
Representative confocal images showing the expression of (**A**) GAD65 or (**B**) GAD67 and PV in the thalamus of epileptic stargazers (STG) and non-epileptic littermates (NE). Merged images at low magnification (10×) and medium magnification (40×) are shown. White and blue dotted boxes in the merged images were magnified in the fourth and fifth panel, respectively. Green boxes in the fourth panel were magnified in the bottom panels of figures (**A**,**B**) to show punctate structures and colocalisation, respectively.

**Figure 4:**
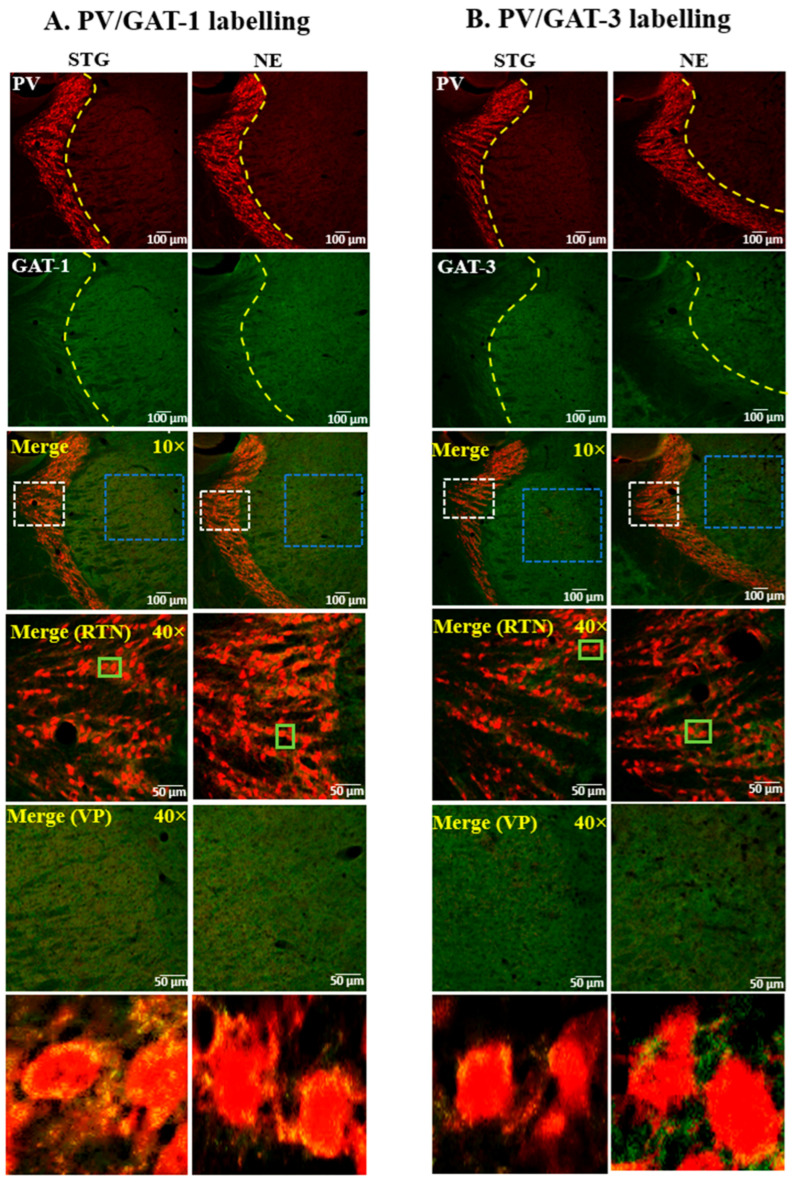
Representative confocal images showing the expression of (**A**) GAT-1 or (**B**) GAT-3 and PV in the thalamus of epileptic stargazers (STG) and non-epileptic littermates (NE). Merged images at low magnification (10×) and medium magnification (40×) are shown. White and blue dotted boxes in the merged images were magnified in the fourth and fifth panel, respectively. Green boxes in the fourth panel were magnified in the bottom panels of figures (**A**,**B**) to show punctate structures.

The expression pattern of GADs and GATs in relation to PV+ interneurons of the SScortex (Figure 1 and Figure 2) and the thalamus (Figure 3 and Figure 4) was examined using confocal immunofluorescence microscopy. Tissue sections from epileptic stargazers and non-epileptic control mice were double labeled with antibodies against PV and either GAD65, GAD67, GAT1, or GAT3. In the SScortex, the soma of PV cells was brightly stained throughout cortical layers 2–6 (Figure 1 and Figure 2) but were most prominent in layer 4, where they were visualised as an intense band (Figure 1 and Figure 2 top row). In the thalamus, the RTN was intensely labelled compared to the VP (Figure 3 and Figure 4), which is consistent with RTN comprising a shell of inhibitory interneurons.

#### 2.1.1. Expression Pattern of GADs in the SScortex

GAD65 expression was uniform throughout the cortical layers appearing as small, punctate staining within the neuropil but not colocalised with PV+ cell bodies, which is consistent with its axon terminal restricted expression (Figure 1A). Magnified merged images showed evidence of some co-labelling of GAD65-positive punctate structures around the edges of PV+ cells (Figure 1A; green box magnified bottom panel). In contrast, GAD67 staining was intensely colocalised with the cell bodies of PV+ interneurons and showed brighter staining in layer 4 where PV+ cell bodies predominantly reside (Figure 1B; green box magnified bottom panel). Overall, the staining pattern of GADs in the SScortex was visually similar for both epileptic stargazer and non-epileptic controls (Figure 1A,B).

#### 2.1.2. Expression of GATs in the SScortex

The expression pattern of GAT-1 and GAT-3 was uniform across SScortex layers with both GATs showing dispersed punctate labelling throughout the SScortex neuropil (Figure 2A,B; green box, magnified bottom panel). GATs were not colocalised with PV soma. Visually, the staining pattern of GAT-1 and GAT-3 in the SScortex was not substantially different between epileptic stargazers and non-epileptic controls (Figure 2A,B).

#### 2.1.3. Expression of GADs in the Thalamus

In the RTN thalamus, GAD65 expression was restricted to punctate structures, likely the axon terminals of GABAergic interneurons. GAD65 staining did not colocalise with the cell bodies of PV+ interneurons, but magnified merged images showed GAD65-positive puncta surrounding PV soma (Figure 3A; green box magnified bottom panel). GAD65 staining was visually similar for both genotypes (Figure 3A). In contrast, GAD67 staining was strongly expressed in the soma of PV+ interneurons in the RTN thalamus of both epileptic stargazers and non-epileptic littermates (Figure 3B; green box magnified bottom panel). In the VP thalamic region, both GADs were uniformly distributed throughout the neuropil (Figure 3A,B; third and fifth panel) with no evidence of labelling within soma. This is consistent with previously published reports [17,39,40] that there are no intrinsic inhibitory neurons within the VP thalamus in rodents. The VP region contains the axonal processes from feed-forward GABAergic interneurons in the RTN projecting onto VP relay neurons. Overall, the staining pattern of GADs in thalamus (RTN and VP) was similar for both epileptic stargazer and non-epileptic controls (Figure 3A,B).

#### 2.1.4. Expression of GATs in the Thalamus

The expression of GAT-1 and GAT-3 was uniform throughout the RTN and VP thalamus (Figure 4A,B). Colocalisation between PV+ soma and GATs was not seen in the VP and RTN thalamus. However, both GATs showed punctate staining around PV-labelled cells in the RTN thalamus (Figure 4A,B, green box magnified bottom panel). Overall, the visual staining pattern of GAT-1 and GAT-3 in the thalamus was not different between epileptic stargazers and non-epileptic controls (Figure 4A,B).

**Figure 5:**
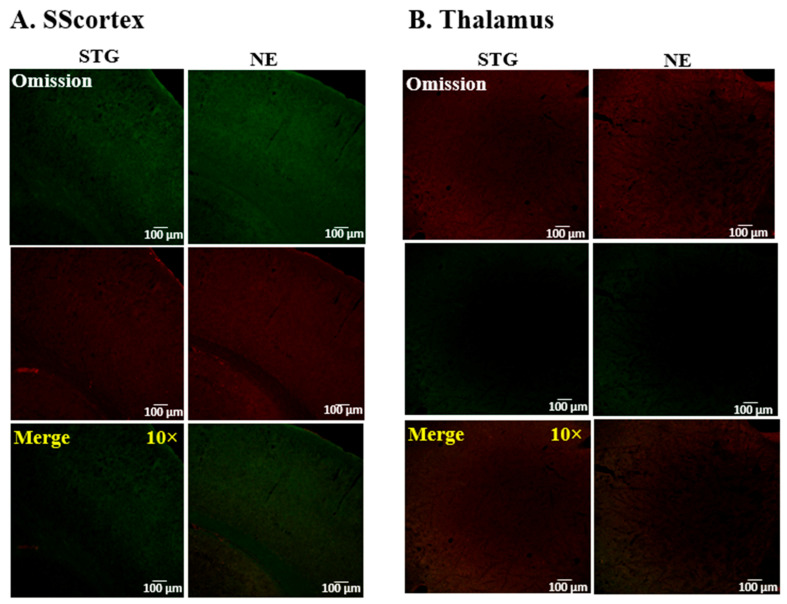
Representative confocal images showing the specificity of secondary antibody binding after the omission of primary antibodies in tissue sections from the (**A**) SScortex and (**B**) thalamus of epileptic stargazers (STG) and non-epileptic littermates (NE). Merged images at low magnification (10×) are shown in the bottom panel.

The omission of primary antibodies eliminated immunolabeling in tissue sections (Figure 5A,B) confirming no non-specific labelling of tissue with the secondary antibodies used in this study.

Together, it can be concluded that the expression patterns of GADs and GATs in the SScortex and the thalamus are not observably different between epileptic stargazers and their non-epileptic counterparts and appear similar to published localisation profiles.

### 2.2. Relative Expression of GADs and GATs in the Tissue Lysates of the SScortex and the VP Thalamus of Stargazers via Western Blotting Analysis

Western blotting analysis was performed to analyse the relative levels of GADs and GATs in whole-tissue lysate of the SScortex and VP thalamus of epileptic stargazers and non-epileptic control littermates. The same antibodies with specificity verified by confocal immunofluorescence were used for semi-quantification of protein levels with Western blotting.

**Figure 6:**
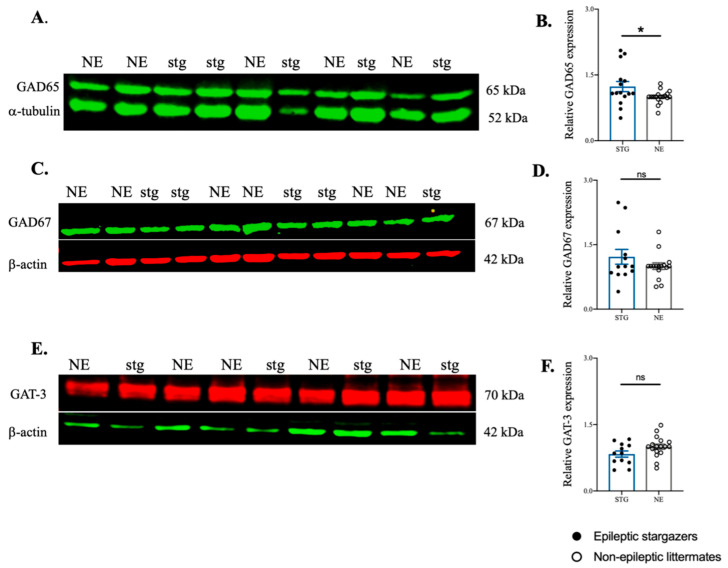
Western blot analysis of GAD65, GAD67, and GAT-3 in the SScortex of stargazers (stg) and non-epileptic littermates (NE). (**A**,**C**,**E**) Representative immunoblots showing the expression of GAD65, GAD67, and GAT-3 with the loading controls α-tubulin or β-actin, respectively. (**B**,**D**,**F**) Relative expression of GAD65, GAD67, and GAT-3 in the SScortex presented as bar graphs. All values represent mean ± SEM. Comparisons were performed using Mann–Whitney unpaired rank test. ns = non-significant, * = *p* < 0.05.

**Figure 7:**
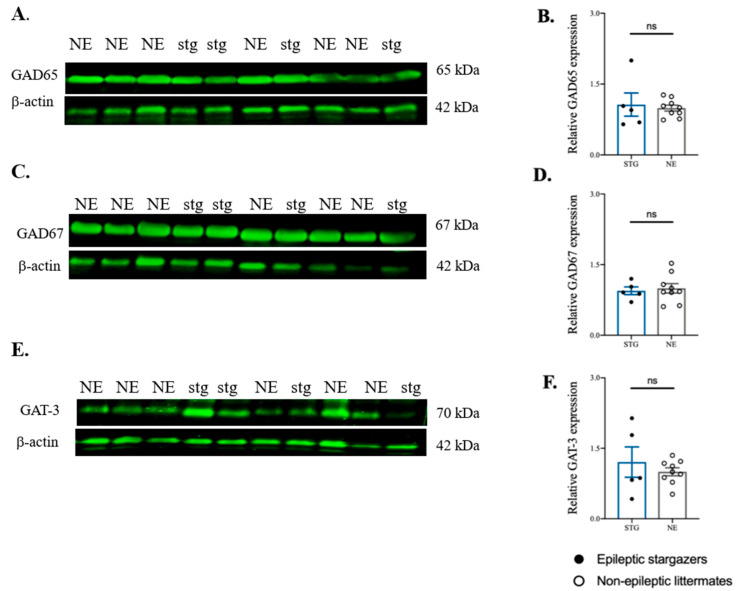
Western blot analysis of GAD65, GAD67, and GAT-3 in the VP thalamus of stargazers (stg) and non-epileptic littermates (NE). (**A**,**C**,**E**) Representative immunoblots showing expression of GAD65, GAD67, and GAT-3 with β-actin. (**B**,**D**,**F**) Relative expression of GAD65, GAD67, and GAT-3 in the VP thalamus presented as bar graphs. All values represent mean ± SEM. Comparisons were performed using Mann–Whitney unpaired rank test. ns = non-significant.

The protein level of GAD65 was significantly increased in SScortex of epileptic stargazers by 24% above their non-epileptic littermates (NE 1.00 ± 0.03, *n* = 18; STG 1.24 ± 0.11, *n* = 15; *p* = 0.024, Figure 6A,B). However, the expression of GAD67 and GAT-3 was not significantly different between genotypes (Figure 6C–F). GAD67 expression trended towards higher levels in epileptic stargazers but did not reach significance (NE 1.00 ± 0.07, *n* = 16; STG 1.217 ± 0.11, *n* = 13, Figure 6C,D). There was no change in GAT-3 expression in epileptic stargazers (0.83 ± 0.07, *n* = 12) compared to non-epileptic littermates (1.00 ± 0.05, *n* = 17) (Figure 6E,F). In the VP thalamus, there were no statistically significant differences in the expression of GAD65, GAD67, and GAT-3 between the genotypes (Figure 7A–F) (GAD65 (NE 1.00 ± 0.03, *n* = 9; STG 1.38 ± 0.30, *n* = 5), GAD67 (NE 1.00 ± 0.07, *n* = 9; STG 0.94 ± 0.11, *n* = 5), GAT-3 (NE 1.00 ± 0.08, *n* = 9; STG 1.61 ± 0.30, *n* = 5)). Unfortunately, GAT-1 was unable to be detected in Western blots using the original antibody or by antibodies from two different suppliers, despite using the highest recommended concentration. Hence, we were unable to analyse the relative levels of GAT-1 in stargazers compared to non-epileptic control littermates.

Overall, Western blotting analysis has shown that GAD65 levels are significantly increased in the SScortex of stargazer epileptic mice. A trend towards heightened levels of GAD67 in the SScortex of epileptic animals, and unaltered cortical levels of GAT-3 have also been demonstrated. None of these protein targets showed a significant difference in VP thalamus expression levels between genotypes.

### 2.3. Expression of GADs and GATs Was Unchanged in Gi-DREADD Animals

To investigate whether the acute functional silencing of FFI in Gi-DREADD animals is sufficient to cause alterations to GADs and GATs expression, Western blotting was performed with whole-tissue lysate of the SScortex of CNO-treated DREADD animals and control counterparts (vehicle-treated DREADD animals and CNO-treated non-DREADD WT animals).

**Figure 8:**
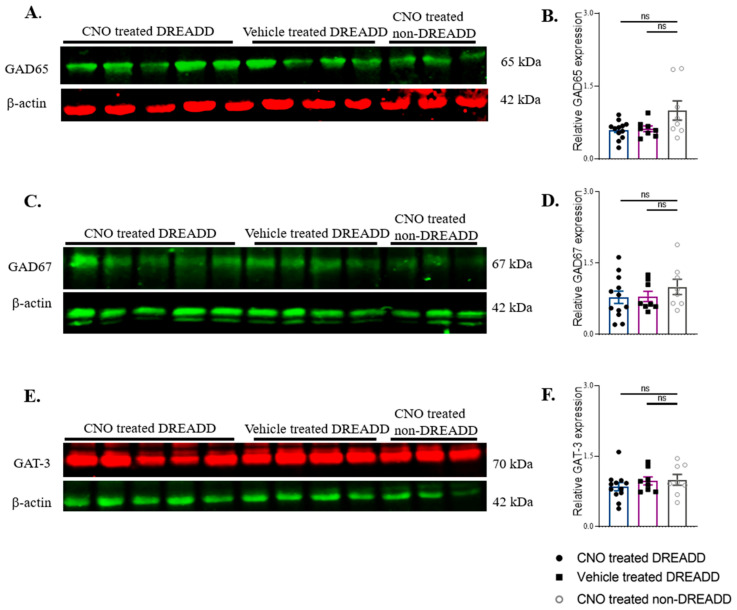
Western blot analysis of GAD65, GAD67, and GAT-3 in the CNO-treated DREADD animals, their vehicle-treated DREADD and CNO-treated non-DREADD control counterparts. (**A**,**C**,**E**) Representative immunoblots showing expression of GAD65, GAD67, and GAT-3 with β-actin. (**B**,**D**,**F**) Relative expression of GAD65, GAD67, and GAT-3 in the SScortex presented as bar graphs. All values represent mean ± SEM. Comparisons were performed using a Mann–Whitney unpaired rank test. ns = non-significant.

Expression levels of GAD65, GAD67, and GAT-3 in the SScortex were not significantly different between groups (Figure 8A–F) (GAD65: CNO-treated DREADD animals (0.60 ± 0.05, *n* = 12); vehicle-treated DREADD (0.62 ± 0.05, *n* = 8); CNO-treated non-DREADD WT control animals (1.00 ± 0.19, *n* = 8)); (GAD67: CNO-treated DREADD animals (0.78 ± 0.12, *n* = 12); vehicle treated DREADD (0.80 ± 0.10, *n* = 8); CNO-treated non-DREADD WT control animals (1.00 ± 0.16, *n* = 8)); (GAT-3: CNO-treated DREADD animals (0.85 ± 0.08, *n* = 12); vehicle-treated DREADD (0.97 ± 0.08, *n* = 8); CNO-treated non-DREADD WT control animals (1.00 ± 0.11, *n* = 8)).

## 3. Discussion

In this study, the impact of loss of FFI on GABA synthesising enzymes (GADs) and GABA transporting proteins (GATs) was investigated. We found that GAD65 levels are significantly increased in the SScortex of epileptic stargazer mice compared to non-epileptic littermates, whereas levels of GAD67 and GAT-3 are unaltered. In contrast, none of these proteins are altered in the VP thalamus of epileptic stargazer mice. Acute administration of CNO into inhibitory Gi-DREADD animals, which silences FFI and is sufficient to generate absence-like seizures, did not change the SScortex expression levels of GAD65, GAD67, or GAT-3. This suggests that changes in GAD65 expression in the stargazer SScortex may occur in response to a sustained reduction of inhibitory activity during development.

### 3.1. Increased Expression of GAD65 in the Stargazer Somatosensory Cortex

Western blotting showed significantly increased GAD65 levels in the SScortex of epileptic stargazer mice. This indicates the excess GABA seen in the SScortex of epileptic stargazers [16] may be due to the increased level of its synthesis enzyme, GAD65. The upregulation of GAD65 may act to increase GABA levels as a compensatory mechanism for the lack of GABAergic FFI in epileptic stargazer mice. As GAD65 predominantly produces GABA for phasic inhibition [21], and FFI is phasic in nature, its upregulation would be a plausible response to the sustained lack of FFI occurring in stargazers. Alternatively, GAD65 upregulation may be occurring early in development, before seizure onset, and contributing to seizure genesis. Excess GABA production by GAD65 could result in more GABA released per synaptic event, and thus stronger GABAergic inhibition and potential spill over to extrasynaptic regions. Such disturbances to phasic, and potentially tonic, inhibition would contribute to CTC disturbances and potentially absence seizure generation or maintenance. GAD65 knockout animals show spontaneous seizures [33,34], but the effect of its upregulation is yet to be determined. Further research tracking GAD65 levels throughout the development of stargazer mice, and genetic or pharmacological manipulation of GAD65 activity, is required to clarify its role in absence seizure genesis. As GAD65 is expressed in axon terminals [41], immunohistochemical labelling showed punctate expression not colocalised with the PV+ soma that form a bright band across layer IV of the cortex [8,42,43]. Uniform GAD65 labelling across the cortical layers indicates its expression in inhibitory axon terminals throughout the SScortex. However, PV+ inhibitory interneurons predominantly synapse onto layer V corticothalamic projection neurons. Thus, further high-resolution immunohistochemical analyses could be used to assess whether GAD65 is upregulated specifically in the axon terminals of PV+ feed-forward inhibitory cells. Alternatively, the network-wide disturbances caused by a lack of FFI may induce compensatory changes in unaffected inhibitory interneuron subtypes, such as somatostatin-positive cells. Such a widespread disturbance may account for the lack of an obvious increase in GAD65 staining in layer V.

In contrast, GAD67, which is expressed throughout cells [13,17,39,40,44,45,46,47], showed intense labelling within the soma of the layer IV PV-labelled cells of the SScortex. However, its levels in the SS cortex were unchanged, as were those of GAT-3, the predominantly neuronal GABA uptake transporter. Although GAT-1 levels could not be quantified to ascertain its contribution to SScortex GABA balance, the observed excess GAD65 is a likely candidate for causing the heightened GABA levels in SScortex of stargazers [16].

### 3.2. Unaltered Expression of GADs and GATs in Gi-DREADD Mice

Proteins can be rapidly synthesised at synapses in response to stimulation levels [38]. To assess whether the SScortex upregulation of GAD65 in stargazers could be a fast response to a lack of activity in FFI neurons, its levels were assessed in an acute model of absence-like seizures, the CNO-treated PV^Cre^/Gi-DREADD animals [14]. However, this model showed no significant differences in the SS cortex expression levels of GAD65, nor of GAD67 or GAT-3. Although Gi-DREADD-mediated silencing of PV+ interneurons is sufficient to generate absence-like seizures, it is insufficient to alter expression of the proteins regulating GABA level. It is possible that Western blotting lacks the sensitivity to detect small, fast changes in GAD65 levels. Alternately, and arguably more likely, the acute nature of Gi-DREADD silencing may not provide a sufficiently prolonged lack of GABAergic inhibition to activate compensatory mechanisms. Thus, the upregulated GAD65 and increased GABA levels observed in stargazers likely requires a sustained lack of feed-forward inhibition to be initiated. Chronic silencing of FFI neurons with Gi-DREADDs may be able to induce GAD65 upregulation, validating this as a response to insufficient FFI. Furthermore, genetic up or downregulation of GAD65 in healthy and absence seizure animals will be able to determine if it is able to induce or exasperate absence seizures.

### 3.3. Unaltered GAD or GAT3 Levels in Stargazer VP Thalamus

In the thalamus, as in the cortex, GAD65 showed a uniform staining pattern across both RTN and VP regions, while GAD67 strongly colocalised with the PV+ soma in RTN but showed uniform labelling in VP. As RTN contains the inhibitory cell bodies that project to VP, GADs in the VP region are those expressed in the axon terminals. Thus, any alterations to GAD expression in this region may be indicative of changes in de novo protein synthesis at the axon terminals [38]. However, we were unable to detect any difference in the VP thalamus expression levels of GAD67 or GAD65 between stargazers and non-epileptic controls using semi-quantitative Western blotting. It is possible that due to the relatively small amounts of protein produced in axon terminals, any changes are too small to be detected using this method. GAT-3 staining was uniform and punctate throughout the neuropil of both RTN and VP thalamus without co-labelling with PV+ soma. VP thalamic GAT-3 levels were also unaltered in stargazers, which is consistent with results observed in GAERS [48].

As no changes in the expression of GADs or GAT-3 were found in VP thalamus, it is likely that these proteins are not responsible for the reduced VP thalamic GABA levels in stargazers [16]. It is possible that this reduction in GABA is a direct consequence of the lack of FFI activity. Region-specific compensatory mechanisms for such a GABA deficit may explain the SScortex-specific upregulation of GAD65 to enhance GABA production and release, while in the VP thalamus, GABA_A_Rs are upregulated to increase postsynaptic sensitivity to GABA release. Further studies assessing the temporal changes in GABA and GAD65 levels across stargazer development will help clarify the drivers of altered GABA levels in different brain regions.

Alternatively, excess GAT-1 in the VP thalamus, causing excess GABA uptake, may be responsible for lowered GABA levels. Unfortunately, this study was unable to quantify changes in GAT-1 levels via Western blotting. As studies have shown the involvement of GAT-1 in absence seizures [26,48], its levels in the stargazer model remain an outstanding question. Previous studies conducted in rats, monkeys, and humans consistently show the expression of GAT-1 in neurons and astrocytes [49], with expression in oligodendrocytes and microglia also reported [50,51]. Thus, it shows a uniform, punctate expression pattern throughout the SScortex and thalamus of stargazers. In the future, quantification of GAT-1 levels in neurons, astrocytes, and other glia of stargazer mice will clarify whether cell type-specific expression changes are occurring in this absence seizure model.

## 4. Materials and Methods

### 4.1. Animals

Animals used in this study were stargazer mice and PV^Cre^/Gi-DREADD mice. Mice were bred and housed at the University of Otago animal facility at controlled room temperature (22–24 °C) with ad libitum access to food and water. All animal procedures were approved by the University of Otago Animal Ethics Committee under the AEC no. DET 32/17 and D94/16.

#### 4.1.1. Breeding Paradigm for Stargazer Mice

Stargazer mice were obtained from Jackson Laboratories, USA. The breeding protocol for stargazer mice in this study was similar to our previously published work [8,9,10]. Heterozygous males were mated with either heterozygous females or homozygous females. The offspring of Het × Het colony were wild-type (+/+), heterozygous (+/stg), and homozygous mutant stargazer (stg/stg) mice, whereas offspring of the Het × Homo colony were +/stg and stg/stg mice. Mice used in this study were adult males aged 2–3 months. For comparative analysis in different experiments, homozygous stargazers (stg/stg) and non-epileptic control littermates (+/+ or +/stg) were used.

#### 4.1.2. Breeding Paradigm for PV^Cre^/Gi-DREADD Mice

PV-Cre knockin and hM4Di-flox mice were obtained from Jackson Laboratories, USA. PV-Cre knockin mice express Cre recombinase in PV+ interneurons without disrupting endogenous parvalbumin expression. hM4Di-flox mice have a *loxP*-flanked STOP cassette designed to prevent transcription of the downstream HA-hM4Di-P2A-mCitrine coding region. The breeding protocol for PV^Cre^/Gi-DREADD mice in this study was similar to our previously published work [14]. We crossed PV-Cre mice with Cre-dependent hM4Di-flox mice to generate littermates with Gi-DREADD receptors selectively expressed in PV+ cells or control mice lacking the Gi-DREADD gene. Cre-mediated removal of STOP cassette allows the strong expression of hemagglutinin (HA)-tagged hM4Di (Gi-DREADD) receptors only in PV cells. Binding of the hM4Di (Gi-DREADD) receptor by the designer drug ‘CNO’ activates the Gi (inhibitory G-protein coupled receptor) pathway, preventing the firing of PV+ cells.

Homozygous female PV-Cre mice were crossed with either homozygous hM4Di-flox males to generate litters of PV^Cre^/Gi-DREADD mice or crossed with heterozygous hM4Di-flox males to generate litters with PV^Cre^/Gi-DREADD and non-DREADD expressing wild-type (WT) control littermates [14]. Only female PV-Cre mice were crossed with male hM4Di-flox mice to avoid unwanted germline recombination. The validation of Gi-DREADD receptor expression in PV cells of PV^Cre^/Gi-DREADD mice was reported in our previous published study [14].

### 4.2. Genotyping

Genotyping was performed to verify the mouse genotypes. Ear notches were collected from the offspring of stargazer and PV^Cre^/Gi-DREADD mice colonies. They were mixed in DNA lysis buffer and proteinase K (Roche, Basel, Switzerland) and digested overnight at 55 °C. The following day, samples were centrifuged and DNA extracted to process for PCR. Genotyping was performed using primers listed in Supplementary Table S1. In PV^Cre^/Gi-DREADD mice, genotyping was performed separately to confirm Cre knockin and hM4Di-flox using Cre, hM4Di mutant, and wild-type primers (Integrated DNA Technologies, Redwood City, CA, USA). The PCR product was run in an agarose gel at 70–80 V for around two hours. Then, the gel was placed on a UV light source to view and photograph bands. Supplementary Figure S1 shows representative genotype bands on an agarose gel for animals of PV^Cre^/Gi-DREADD and stargazer colonies.

### 4.3. Immunofluorescence Confocal Microscopy

Adult stargazer mice (epileptic stargazers *n* = 4; and non-epileptic controls *n* = 3) were deeply anesthetised with an intraperitoneal injection of 60 mg/kg of sodium pentobarbital. Then, transcardial perfusion was performed with 5% heparin in 0.1 M phosphate-buffered saline (PBS), which was followed by 4% paraformaldehyde (PFA) in 0.1 M Sorensen’s phosphate buffer (PB). Brains were carefully extracted and post-fixed in 4% PFA overnight at 4 °C. The next day, brains were washed three times in 0.1 M PB followed by cryoprotection in increasing concentration of sucrose in PBS, i.e., 10% for 30 min, 20% for 30 min, and 30% at 4 °C until the brains sunk to the bottom of the glass container. Brains were sectioned into 30 µm coronal sections on a freezing cryostat (Leica CM1950, Wetzlar, Germany), and sections were collected into 12-well plates containing PBS.

Sections were transferred into blocking buffer (4% Normal Goat Serum (NGS), 0.1% Bovine Serum Albumin (BSA), 0.1% Triton X-100 in PBS) for two hours at room temperature. Following blocking, sections were incubated with the appropriate primary antibodies diluted in PBS with 0.1% BSA and 0.3% Triton X-100 for 48 h at 4 °C. Primary and secondary antibodies used for immunofluorescence confocal microscopy are listed in Table 1. Then, tissue sections were washed in PBS three times for 15 min each before incubation with appropriate secondary antibodies diluted in PBS for approximately 12 h at 4 °C. This was followed with a further three, ten-minute PBS washes prior to mounting on polysine-coated glass slides and cover-slipping with mounting medium (1,4 diazabicyclo (2.2.2) octane DABCO-glycerol). Slides were air-dried in the dark at room temperature.

### 4.4. Confocal Image Acquisition

Images were acquired using a Nikon A1+ inverted confocal laser scanning microscope. Channel configurations were set for the green channel (488 nm laser excitation) and red channel (568 nm laser excitation). During confocal imaging, the detector offset for each channel was kept at zero; the detector gain, laser power, scan speed, and image pixel size were optimised accordingly. Images were captured of the region of interest (ROI) in the SScortex and thalamus (RTN and VP) in sections from the brain.

### 4.5. Western Blotting

Animals were sacrificed by cervical dislocation. Brains were immediately extracted, snap-frozen on dry ice, and stored at −80 °C. The cerebellum was dissected from the rest of the brain and forebrain mounted in the cryostat chuck using embedding media (Optimal cutting temperature compound (OCT compound)) (Leica Microsystems, Buffalo Grove, IL, USA). The temperature inside the cryostat chamber was always maintained at −10 °C. Then, 300 µm thick coronal sections were cut and thaw-mounted on glass slides. Slides were temporarily stored on dry ice to prevent degradation of the tissues. The primary SScortex and VP thalamus were identified using the Mouse Brain Atlas (Paxinos & Franklin, 3rd edition) and punched out from sections with 1.0 mm biopsy punches (Integra Miltex, 33–31AA) under a dissecting microscope. Micro-punched tissues were collected into microtubes containing homogenisation buffer (0.5M Tris, 100 mM EDTA, 3% SDS, pH 6.8), 1% phenylmethylsulfonyl fluoride (PMSF), and 1% protease inhibitor (P8340, Sigma, Livonia, MI, USA). Samples were sonicated for five minutes, heated for two minutes at 100 °C, and then centrifuged at 13,000 rpm for five minutes at 4 °C. The supernatant from each microtube was transferred into a new microtube and stored at −80 °C until use.

In the stargazer-based Western blotting experiments, 27 epileptic stargazers and 31 non-epileptic control animals from 20 litters were included. In the DREADD-based Western blotting experiments, 12 CNO-treated DREADD animals, 8 vehicle-treated DREADD animals, and 8 CNO-treated non-DREADD WT controls were used. In the DREADD experiment, animals were injected with CNO/vehicle (10 mg/kg) and sacrificed within 30 min after injection Timing of the sacrifices was based on results from our previous published work [14] where silencing PV+ interneurons generated absence-like seizures within 30 min of CNO injection. Tissue lysates were analysed using appropriate antibodies to each protein target. The primary and secondary antibodies used for this experiment are listed in Table 1. Protein bands were photographed on a Li-Cor Odyssey Scanner and analysed using Odyssey Image Studio Lite v3.1. Protein of interest bands were normalised to the intensity of the loading control (β-actin or α-tubulin) band in the same lane.

### 4.6. Preparation of CNO

First, 1.5 mg of CNO (Advanced Molecular Technologies, Scoresby, Australia) was dissolved in 75 μL of dimethyl sulfoxide (DMSO). Then, the volume was adjusted to 1.5 mL by the addition of 0.9% sterile saline to prepare CNO of 1 mg/mL concentration. Vehicle was prepared by mixing DMSO with 0.9% sterile saline. CNO or vehicle was injected intraperitoneally at 10 mg/kg. For focal injections, 0.3 μL of CNO was infused into the specific brain region at a rate of 0.1 μL/min. It was delivered via a Hamilton microinjection syringe attached to polythene tubing (Microtube Extrusions, North Rocks, Australia) and a 33-gauge internal cannula (Plastics One Inc., Roanoke, VA, USA) inserted into the previously implanted guide cannula.

### 4.7. Statistical Analysis

Statistical analyses were performed in GraphPad Prism 9.1.1. Data are presented as mean ± standard error of the mean (SEM). Statistical differences in the expression levels of protein in the Western blotting experiments were tested using the Mann–Whitney unpaired rank test. Statistical significance was set at *p* < 0.05 (asterisks for *p* value: * *p* < 0.05, ** *p* < 0.01, *** *p* < 0.001, and **** *p* < 0.0001).

## 5. Conclusions

In the stargazer mouse model of genetic absence epilepsy, FFI neurons are less responsive to activation. The upregulation of GAD65 observed in this study may be induced as an attempt to compensate for a chronic lack of FFI, with acute silencing of FFI insufficient to trigger such a transcriptional response. Excess GAD65 would increase GABA synthesis at axon terminals, thus resulting in more GABA released per synaptic event, heightening SScortex GABA levels, as has been reported in stargazers [16]. Excess GABA release would result in stronger inhibitory input to downstream excitatory neurons, despite the reduced frequency of inhibitory firing. This may further disrupt the CTC network activity, exasperating seizures. If this is this case, pharmacological modulation of GAD65 levels or activity may be a useful therapy for CAE patients for whom current treatments exasperate seizures. Additionally, a compensatory upregulation of GAD65 may explain the exasperated seizures experienced by some patients when treated with drugs to dampen GABA activity. The discovery that GAD65 is upregulated in the SScortex of the stargazer model of absence epilepsy has identified a novel and potentially crucial factor in the etiopathology of absence seizures.

## Figures and Tables

**Table 1:** Primary and secondary antibodies used in this study for immunofluorescence confocal microscopy and Western blotting.

Product	Antibody/Type	Source/Catalogue No.	RRID	Dilution
**Immunofluorescence Confocal Microscopy**				
Parvalbumin	Primary/Mouse monoclonal	Swant/235	AB_10000343	1:2000
Parvalbumin	Primary/Rabbit polyclonal	Swant/PV27	AB_2631173	1:2000
GAT-1	Primary/Rabbit polyclonal	Abcam/ab426	AB_2189971	1:500
GAT-3	Primary/Rabbit polyclonal	Alomone Labs/AGT-003	AB_2340977	1:200
GAD 65	Primary/Mouse monoclonal	Abcam/ab26113	AB_448989	1:500
GAD 67	Primary/Mouse monoclonal	Millipore/MAB5406	AB_2278725	1:500
Goat anti-rabbit	Secondary/Alexa Fluor 488	Life Technologies/11008	AB_143165	1:1000
Goat anti-mouse	Secondary/Alexa Fluor 568	Life Technologies/11031	AB_144696	1:1000
**Western blotting**				
β-actin	Primary/Mouse monoclonal	Abcam/ab8226	AB_306371	1:1000
β-actin	Primary/Rabbit monoclonal	Cell Signalling/4970	AB_2223172	1:1000
α-tubulin	Primary/Rabbit polyclonal	Abcam/ab4074	AB_2288001	1:5000
GAT-1	Primary/Rabbit polyclonal	Abcam/ab426 Alomone Labs/AGT-001	AB_2189971	1:250–500 1:200
GAT-3	Primary/Rabbit polyclonal	Alomone Labs/AGT-003	AB_2340977	1:200
GAD65	Primary/Mouse monoclonal	Abcam/ab26113	AB_448989	1:750
GAD67	Primary/Mouse monoclonal	Millipore/MAB5406	AB_2278725	1:2500
Goat anti-rabbit	Secondary/IRDye 680	Li-Cor/926-32221	AB_621841	1:10,000
Goat anti-rabbit	Secondary/IRDye 800 CW	Li-Cor/926-32211	AB_621843	1:10,000
Goat anti-mouse	Secondary/IRDye 800 CW	Li-Cor/926-32210	AB_621842	1:10,000

## Data Availability

Data is accessible upon reasonable request.

## Supplementary Materials

The following are available online at https://www.mdpi.com/article/10.3390/ijms22147740/s1, Figure S1: Representative images of genotype bands on agarose gel. (A) Images showing the homozygous (Homo) PV-Cre knockin (350 bp and 300 bp) and the hM4Di-flox for four mice to verify PV-Cre knockin and hM4Di-flox [for hM4Di-flox: heterozygous (het) 300 bp and 204 bp; wild-type (WT) 300 bp] (B) Images showing the bands for wild-type (+/+) mice at 360 bp, heterozygous (+/stg) mice with two bands at 360 bp and 155 bp, and epileptic stargazers (stg/stg) with a band at 155 bp, Table S1: Primers used for genotyping of PVCre/Gi-DREADD and stargazer mice.

## Abbreviations

AEDs: anti-epileptic drugs; AMPA: α-amino-3-hydroxy-5-methyl-4-isoxazolepropionic acid; CAE: childhood absence epilepsy; CNO: clozapine-N-Oxide; CTC: cortico-thalamocortical; DREADDs: designer receptors exclusively activated by designer drug; EEG: electroencephalogram; ETX: ethosuximide; FFI: feed-forward inhibition; GABA: gamma-aminobutyric acid; GAD: glutamic acid decarboxylase; GAERS; genetic absence epilepsy rat from Strasbourg; GAT: GABA transporter; NE: non-epileptic; OCT: optimum cutting temperature; PB: phosphate buffer; PBS: phosphate-buffered saline; PFA: paraformaldehyde; PV+: parvalbumin expressing; ROI: region of interest; RTN: reticular thalamic nuclei; SEM: standard error mean; SScortex: primary somatosensory cortex; STG: stargazer; SWDs: spike-wave discharges; VP: ventral posterior: WT: wild-type.
