# Supplementary material for: Impact of Dysfunctional Feed-Forward Inhibition on Glutamate Decarboxylase Isoforms and γ-Aminobutyric Acid Transporters
Source: Int J Mol Sci. 2021 Jul 20;22(14):7740. doi: 10.3390/ijms22147740 (PMC8306481; doi:10.3390/ijms22147740)
Supplement: Supplementary file 1 [file ijms-22-07740-s001.zip › ijms-1297450-supplementary.pdf]

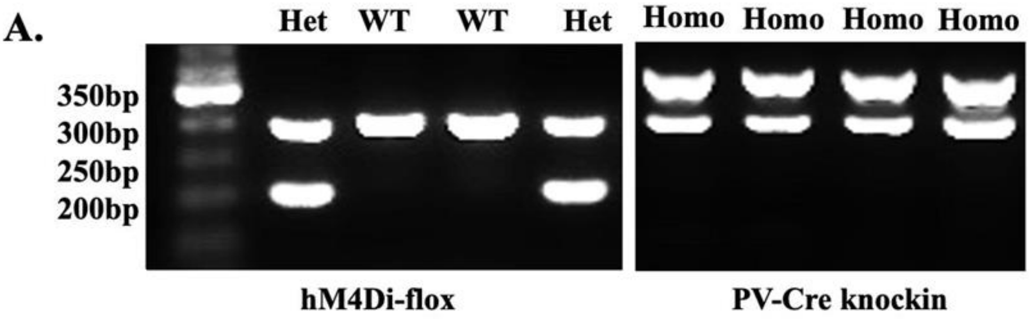

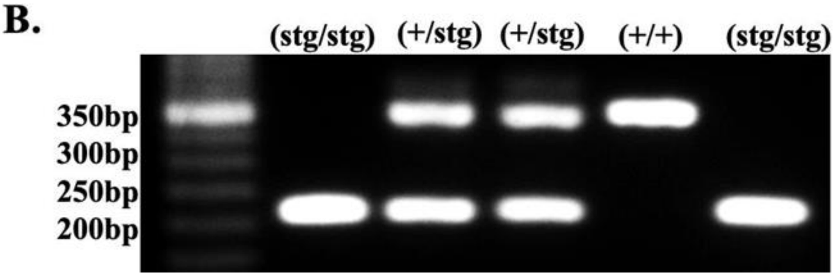

Supplementary Fig. S1 Representative images of genotype bands on agarose gel. (A) Images showing the homozygous (Homo) PV-Cre knockin (350bp and 300bp) and the hM4Di-flox for four mice to verify PV-Cre knockin and hM4Di-flox [for hM4Di-flox: heterozygous (het) 300 bp and 204 bp; wild-type (WT) 300 bp] (B) Images showing the bands for wild-type (+/+) mice at 360 bp, heterozygous (+/stg) mice with two bands at 360 bp and 155 bp, and epileptic stargazers (stg/stg) with a band at 155 bp.

Table S1. Primers used for genotyping of PV^{Cre}/Gi-DREADD mice and stargazer mice.

**Primer type**	**Sequence (5′→3′)**
**PV^{Cre}/Gi-DREADD mice**	
hM4Di mutant forward	CGA AGT TAT TAG GTC CCT CGA C
hM4Di mutant reverse	TCA TAG CGA TTG TGG GAT GA
Wild type forward	AAG GGG CTG CAG TGG AGT A
Wild type reverse	CCG AAA ATC TGT GGG AAG TC
Cre forward	CCT GGA AAA TGC TTC TGT CCG
Cre reverse	CAG GGT GTT ATA AGC AAT CCC
	**Stargazer mice**
Common forward	TAC TTC ATC CGC CAT CCT TC
Wild type reverse	TGG CTT TCA CTG TCT GTT GC
Mutant reverse	GAG CAA GCA GGT TTC AGG C
